# Epidemiological Analysis of SARS-CoV-2 Transmission Dynamics in the State of Odisha, India: A Yearlong Exploratory Data Analysis

**DOI:** 10.3390/ijerph182111203

**Published:** 2021-10-25

**Authors:** Sourya Subhra Nasker, Ananya Nanda, Balamurugan Ramadass, Sasmita Nayak

**Affiliations:** 1 School of Biotechnology, Kalinga Institute of Industrial Technology, Bhubaneswar 751024, Odisha, India; souryasubhra96@gmail.com (S.S.N.); ananya1992@gmail.com (A.N.); 2 All India Institute of Medical Sciences, Bhubaneswar 751019, Odisha, India; biochem_ramadass@aiimsbhubaneswar.edu.in; 3 Kalinga Institute of Medical Sciences, Kalinga Institute of Industrial Technology, Bhubaneswar 751024, Odisha, India

**Keywords:** SARS-CoV-2, COVID-19, epidemiological surveillance, temperature effect, population-based studies, prevention, risk assessment

## Abstract

COVID-19 remains a matter of global public health concern. Previous research suggested the association between local environmental factors and viral transmission. We present a multivariate observational analysis of SARS-CoV-2 transmission in the state of Odisha, India, hinting at a seasonal activity. We aim to investigate the demographic characteristics of COVID-19 in the Indian state of Odisha for two specific timelines in 2020 and 2021. For a comparative outlook, we chose similar datasets from the state of New York, USA. Further, we present a critical analysis pertaining to the effects of environmental factors and the emergence of variants on SARS-CoV-2 transmission and persistence. We assessed the datasets for confirmed cases, death, age, and gender for 29 February 2020 to 31 May 2020, and 1 March 2021 to 31 May 2021. We determined the case fatalities, crude death rates, sex ratio, and incidence rates for both states along with monthly average temperature analysis. A yearlong epi-curve analysis was conducted to depict the coronavirus infection spread pattern in the respective states. The Indian state of Odisha reported a massive 436,455 confirmed cases and 875 deaths during the 2021 timeline as compared to a mere 2223 cases and 7 deaths during the 2020 timeline. We further discuss the demographic and temperature association of SARS-CoV-2 transmission during early 2020 and additionally comment on the variant-associated massive rise in cases during 2021. Along with the rapid rise of variants, the high population density and population behavior seem to be leading causes for the 2021 pandemic, whereas factors such as age group, gender, and average local temperature were prominent during the 2020 spread. A seasonal occurrence of SARS-CoV-2 transmission is also observed from the yearlong epidemiological plot. The recent second wave of COVID-19 is a lesson that emphasizes the significance of continuous epidemiological surveillance to predict the relative risk of viral transmission for a specific region.

## 1. Introduction

A cluster of pneumonia cases of unknown etiology was identified in the Huanan seafood wholesale market in Wuhan, China, in late December 2019 [1,2,3,4,5,6]. By 7 January 2020, the Chinese authorities had identified and isolated a novel coronavirus. Due to this, other respiratory viruses such as influenza virus, adenovirus, SARS-CoV, and MERS-CoV were eliminated as the potential cause. The International Committee on Taxonomy of Viruses termed the novel coronavirus as SARS-CoV-2, and the World Health Organization (WHO) named it Coronavirus Disease 2019 (COVID-19) [7,8,9]. On 11 March 2020, the WHO declared the COVID-19 outbreak as a global pandemic [10]. Since its emergence in Wuhan, China, SARS-CoV-2 has quickly spread across every continent claiming the lives of around 4 million people worldwide [11]. Our current knowledge of COVID-19 comes predominantly from continuous disease surveillance and epidemiological investigations conducted from the early phase of the pandemic in China, Europe, and North America to daily reports of viral dissemination across the globe.

COVID-19 has been identified as an aerosolized infection where viral entry is mediated by the SARS-CoV-2 spike (S) protein via host angiotensin-converting enzyme 2 (ACE2) receptors [12,13,14,15]. Studies have further reinforced an inverse relationship of temperature and humidity with SARS-CoV-2 transmission [16,17,18,19,20,21]. Byun et al. have talked about a seasonal variation of COVID-19 occurrence due to temperature sensitivity [22]. During the initial half of the pandemic in 2020, the likelihood of acquiring COVID-19 involved demographic as well as meteorological factors. However, as the pandemic progressed, viral persistence led to the emergence of new variants with mutations likely to confer a fitness advantage [23].

Variants of concern or VOCs, as classified by the National Center for Immunization and Respiratory Diseases (NCIRD), Division of Viral Diseases, and published by the CDC, include four major variants, Alpha, Beta, Gamma, and Delta [23,24]. These variants have gathered mutations encompassing the S protein region, altering the flexibility of the spike, allowing better entry via ACE2 or other receptors and aiding in immune escape [25,26]. The first mutation D614G in the receptor-binding domain (RBD) of S protein was identified in Europe in late February 2020. Global transition from the original D614 to variant G614 occurred in mid-May 2020 [27]. The Beta variant with an N510Y mutation was identified in South Africa during May 2020 and had further spread dominantly in the Indian subcontinent [28]. By September 2020, an Alpha variant was reported in the UK that gathered a cluster of 23 mutations in the S protein region. During that time, 54 Indian isolates have been monitored with the Alpha-variant-specific P681H [26,29]. The Delta variant with 15 spike substitutions originated in India during October 2020. As of January 2021, Delta isolates with A222V and S447N mutations were noticeable in India [24,26]. Mid-December 2020 to early January 2021, the Gamma variant was identified in Brazil with a cumulative 17 amino acid mutations [26]. In the light of recent events, Delta is now the most common variant in India, accounting for more than 90% of new infections and over 20% of new cases in the United States of America [26,30].

India documented its first COVID-19 case on 30 January 2020, in Kerala. In a matter of 47 days, Odisha, the 11th largest state of India in terms of population reported its index case. From May 2020 onward, the state of Odisha started reporting around 1300 average daily cases till the end of 2020. The first wave of Odisha started in June 2020 lasting until November 2020 with around 1700 average daily cases. The first COVID-19 wave in Odisha declined by early December 2020, only to drastically rise from April 2021 with a daily average of around 7000 cases. Odisha is currently going through the second wave of the COVID-19 pandemic with 912,887 cumulative confirmed cases and 4063 cumulative deaths until July 2021 [31].

In the current work, we report a contrast in SARS-CoV-2 spread in the state of Odisha (OD), India, during the years 2020 and 2021 where we correlate demographic parameters such as age group distribution of cases, case fatality rate (CFR), crude death rate (CDR), sex ratio, and incidence rate along with a temperature analysis. For our study, we chose similar timelines for the year 2020 (29 February 2020 to 31 May 2020) and 2021 (1 March 2021 to 31 May 2021). For a comparative analysis, we gathered information on similar datasets for the state of New York (NY), USA, keeping the timelines conserved for 2020 and 2021. We further constructed a combined yearlong epidemiological curve analysis using time-series confirmed case datasets, to understand the transmission dynamics of SARS-CoV-2 and variants across OD and NY from 29 February 2020 to 31 May 2021.

## 2. Methods

### 2.1. Study Design and Data Sources

The study design is based on exploratory data analysis using statistical graphs to analyze the cases of COVID-19 observed in OD and draw a comparison with NY. The period of study for the year 2020 includes cases and deaths numbers from 29 February 2020 to 31 May 2020 for both states, detailed in Table 1. For the year 2021, specifically, the cases and deaths of both states were included from 1 March 2021 to 31 May 2021, represented in Table 2. The study is depicted as a cross-sectional cohort design, and hence we used the STROBE Guidelines [32] to aid our thorough comparison between the two states in terms of observational research.

For 2020, the case records for OD were collected from COVID Dashboard, an official website launched by the Government of Odisha [31] by monitoring daily cases meticulously. However, the case records for NY were collected from the official website of the New York State Department of Health [33]. For 2021, the case records for OD were collected from the raw data sources of COVID Dashboard [31], Government of Odisha, whereas GitHub contained all the time-series datasets for NY [34]. Neither was any sampling performed to determine a sample space nor was any eligibility criterion for considering particular cases established—all cases were taken.

### 2.2. Analysis

We constructed age distribution graphs by converting the confirmed case and death case numbers into percentages of total cases observed for OD and NY in 2020. Gender distribution graphs were also constructed similarly for both states along with the calculation of the male:female (M:F)ratio for 2020. For the two years, case fatality rates for both states were calculated individually as the total number of deaths divided by the total number of cases, expressed in percentage. Crude death rates for both states were also calculated individually as the total number of deaths divided by the total number of projected populations in 2020 and 2021, expressed per 100,000 of the population. The projected population data were collected from the official websites of the United States Census Bureau and the Office of the Registrar General and Census Commissioner, India [35,36]. Incidence rates were calculated for the two years by dividing the total number of cases with the projected population each year, expressed per 1000 population.

Our study also provides an investigation to ascertain the role of geophysical indicators on COVID-19 transmission. We depict a time-series plot with monthly maximum, minimum, and average temperature for each state from February 2020 to May 2021. A combined yearlong epidemiological investigation was conducted by plotting the number of confirmed cases (y-axis) versus the date of symptom onset (x-axis) to understand the progression of disease in both states from 29 February 2020 to 31 May 2021, additionally commenting on the first and second waves of COVID-19 for each state.

## 3. Results

### 3.1. Confirmed Cases, Death Cases, Case Fatality Rates, and Crude Death Rates for 2020 Timeline

Table 1 depicts the confirmed cases, death cases, CFR, and CDR for the 2020 timeline in OD and NY. A total of 2223 confirmed cases and only 7 deaths with an overall CFR of 0.3% were found for OD. The confirmed cases in OD started to peak from May 2020 onward, contributing to 93.1% of the total cases included in 2020. For the same timeline, NY reported a maximum number of cases and deaths with 203,006 confirmed and 17,766 deaths with an overall CFR of 8.8%. A rapid spread was observed in NY starting in March 2020 and peaking until April 2020, and it started to decline by May 2020. The CDR was overall visibly higher for NY than for OD with most deaths occurring in April 2020.

**Figure 1:**
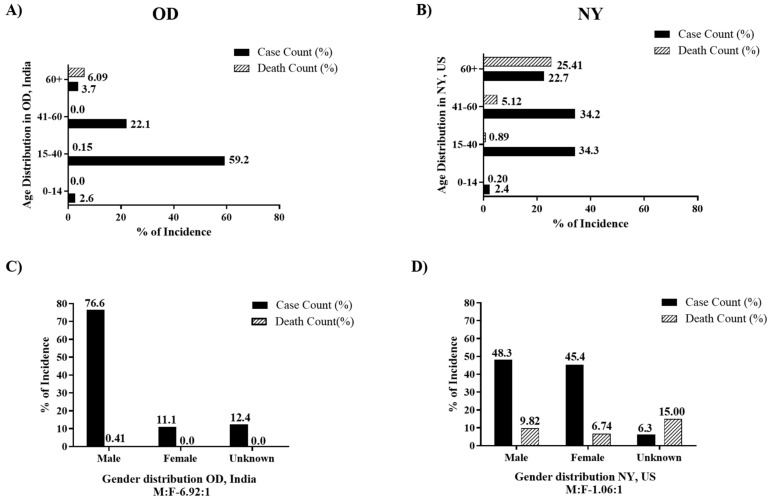
Age and gender distribution of COVID-19 cases from 29 February 2020 to 31 May 2020. Percentage of disease incidence within different age groups in (**A**) OD and (**B**) NY. Percentage of disease incidence within different gender in (**C**) OD and (**D**) NY. Sex ratio (i.e., male-to-female (M:F) ratio) is shown below each graph. The graph is plotted by converting case numbers to percentages of the total number to maintain the scale. Death count (%) is calculated by dividing the death cases in each age group by the corresponding confirmed case in that age group. Abbreviations: OD, State of Odisha, India. NY, State of New York, USA.

Table 1 also depicts the age and gender distribution of confirmed and death cases with individual CFR and CDR for the 2020 timeline in OD and NY, as represented in Figure 1. The age group 15–60 years contributed to most of the confirmed cases in OD (Figure 1A), with a total of seven deaths in the state, five were reported for individuals ≥60 years with a CFR of 6.1%. An increase in the number of confirmed cases in males was observed in OD. All seven deaths in OD occurred in male patients. No female deaths were reported till May 2020. The male to female ratio (M:F) was calculated to be 6.92:1 (Figure 1C).

Individuals between 15–60 years also contributed the most to the confirmed case count in NY followed by those who were 60+ years (Figure 1B). The total death cases contributed by age above 60 years was the highest with a CFR of 25.4%. The gender distribution of confirmed cases in NY appears to be almost similar between males and females with an (M:F) ratio of 1.06:1 (Figure 1D). Although there was a similar risk of infection for both genders in NY, the male population was more susceptible to severe disease and subsequent death with higher CFR and CDR than females (Table 1).

### 3.2. Confirmed Cases, Death Cases, Case Fatality Rates, and Crude Death Rates for 2021 Timeline

Table 2 represents the confirmed cases, death cases, CFR, and CDR for the 2021 timeline in OD and NY. There was a considerable increase in confirmed case numbers for OD with 875 deaths accounting for a mere 0.2% CFR. The case numbers started to rise from April 2021 with more than 100,000 cases and lasted throughout the month of May 2021 (319,125 cases). Cumulative case numbers in NY appeared to be much lower than in OD, although NY reflected a surge in case numbers before March 2021 as seen from Table 2.

Age- and gender-related datasets for 2021 could not be retrieved completely due to limitations in accessing the public domain raw data template. The cause of the overall high CFR and CDR values in NY than in OD in 2021, as well as 2020, was rather inconclusive. Hence, we calculated the incidence rates for each timeline to support our analysis.

### 3.3. Incidence Rates for 2020 and 2021 Timelines

The incidence rates were calculated for both states individually for the timelines 2020 and 2021 as explained in the Methods section (Section 2). For 2020, the incidence rates were 0.05% and 10.42% for OD and NY, respectively, even though the population of OD is roughly twice that of NY. In 2021, the incidence rates were calculated as 9.2% and 7.8% for OD and NY, respectively. A higher percentage in incidence rate suggests an intensified viral spread in OD during 2021.

### 3.4. Yearlong Temperature Analysis

**Figure 2:**
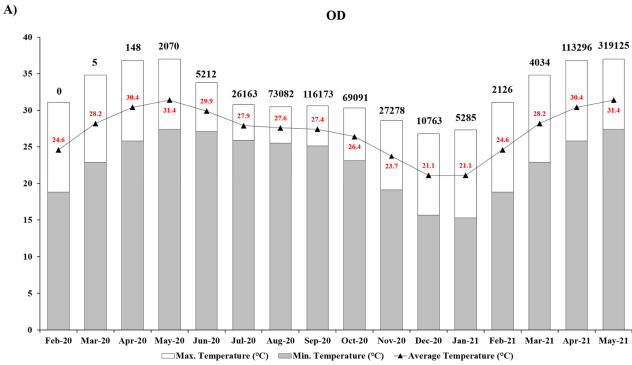

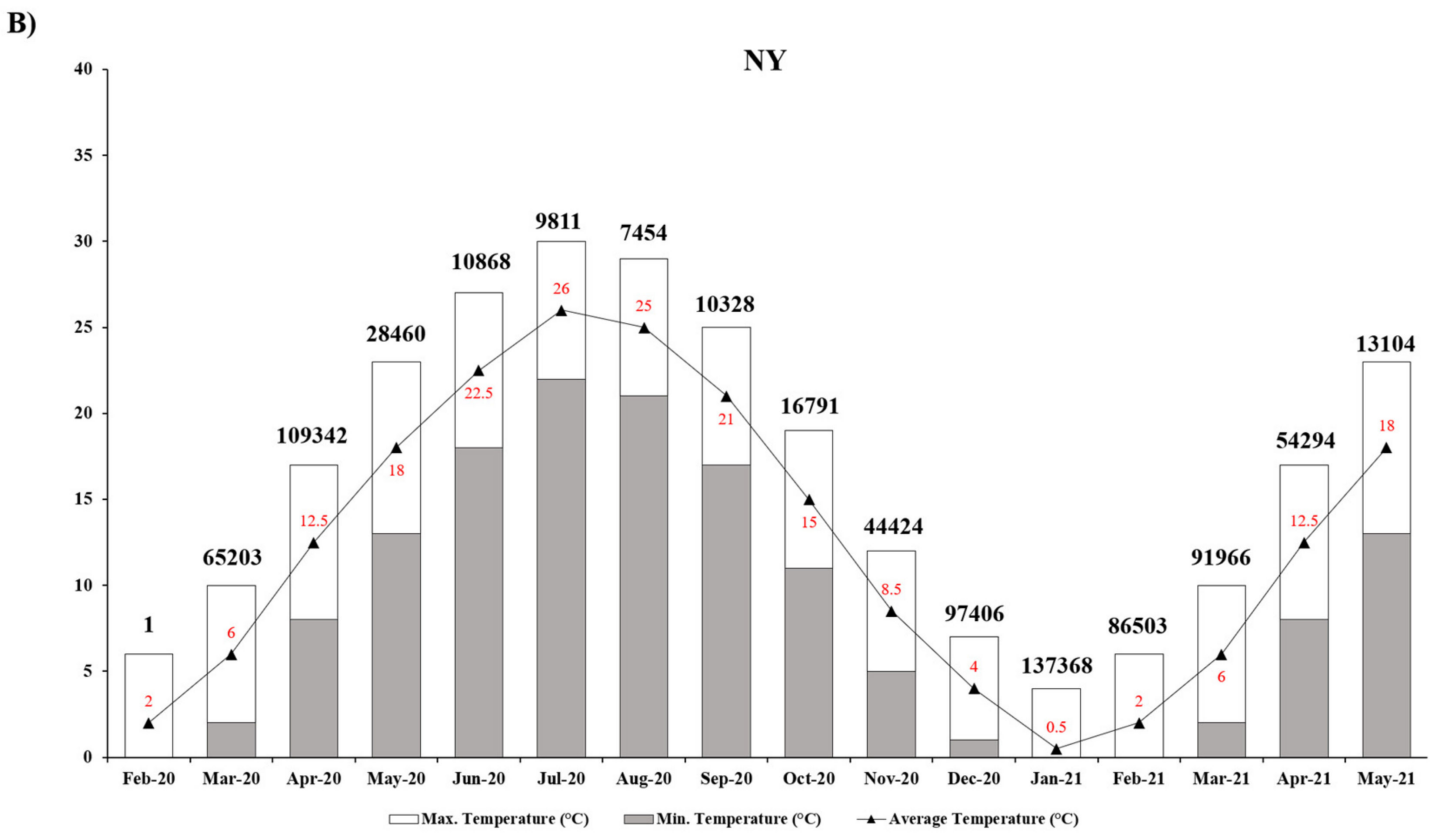
Graphical representation of monthly average surface temperature in (**A**) OD and (**B**) NY from February 2020 to May 2021. Confirmed case numbers for individual months are shown above each bar graph. Abbreviations: OD, State of Odisha, India. NY, State of New York, USA.

The monthly average, maximum, and minimum temperatures are plotted from February 2020 to May 2021 in Figure 2(A(OD),B(NY)). We have considered only the monthly average temperatures for our study. For further correlation, cumulative confirmed case numbers are shown above each temperature graph. OD faced high monthly average temperatures during the early phase of the SARS-CoV-2 pandemic. The case numbers for COVID-19 started to rise from May 2020 (Table 1), coinciding with a fall in the monthly average temperature in OD till January 2021 (Figure 2A). The average temperature increased in OD after January 2021 correlating with low case numbers during this period (Table 2).

NY, having a temperate climate, recorded average temperatures below 13 °C until April 2020, concurrent with the highest number of confirmed cases during this time (Table 1). A regression in case numbers in NY was noticed (Table 1) that coincided with the rise in the average temperature from May 2020 (Figure 2B). NY experienced a lower monthly average temperature (below 10 °C) during November 2020 and through to March 2021. A yearlong epidemiological curve analysis provided a correlation of observed temperature variation with the occurrence of first and second waves of COVID-19 in both states as explained in the upcoming section (Discussion, Section 4).

### 3.5. Yearlong Epidemiological Curve Analysis of COVID-19 Confirmed Cases

**Figure 3:**
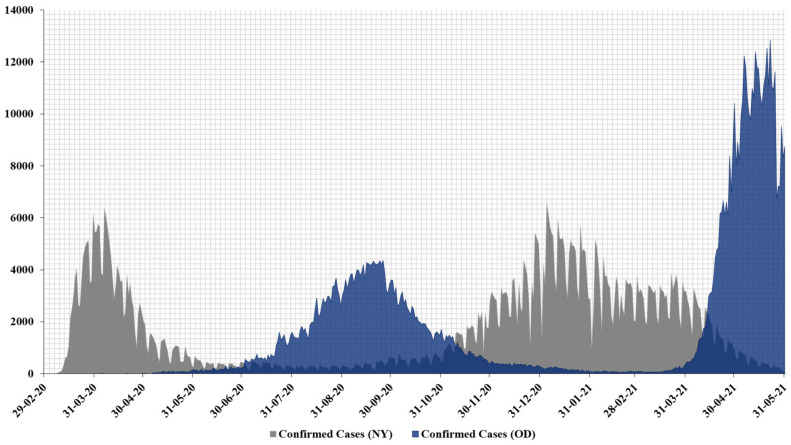
A yearlong epidemiological curve from 29 February 2020 to 31 May 2021, showing the first wave and second wave of COVID-19. The figure depicts the COVID-19 outbreak trend in the state of OD (blue) and NY (gray) during the aforementioned timeline. Abbreviations: OD, State of Odisha, India. NY, State of New York, USA.

The epidemiological curve depicting the time series data for confirmed cases in OD is shown in Figure 3. Since its occurrence on 16 March 2020, the progression of COVID-19 in OD was rather delayed during the initial months. Confirmed case numbers started to rise in June, peaking in September 2020 with 4356 cases, and declining by November 2020, indicating the first wave. After a gap of 4 months, the second wave of COVID-19 devastated OD with cumulative daily cases going as high as 12,852 on 22 May 2021, alone. The second wave in OD was still ongoing during the current study.

In a side-by-side comparison, the yearlong epi-curve for NY is also combined in Figure 3. Confirmed case numbers for the first wave started to rise increasingly since its index case on 1 March 2020, lasting through the next month with 4510 peak confirmed cases. After 6 months of controlled case numbers, the second wave in NY initiated later the same year in November 2020, peaking on 4 January 2021, with 6578 cases and declining by April 2021.

From the combined epidemiological curve, the pattern of the first and second waves can be made out, suggesting a debilitated viral spread in OD compared to the rapid dissemination of SARS-CoV-2 in NY during the first wave (Year 2020). The second wave in OD also seem to get delayed (December 2020–February 2021), but after its initiation, the second wave peak of OD was almost three times that of its first wave peak. NY managed to keep the second wave’s daily confirmed case numbers at par with its first wave numbers. The duration of first and second waves in the respective states also hints at a seasonal activity for SARS-CoV-2 similar to that of the influenza season in the Indian subcontinent and the United States [37,38].

## 4. Discussion

Our findings, based on epidemiological surveillance of demographic and temperature data from the Indian state of Odisha and the state of New York, USA, provides insight into the difference in SARS-CoV-2 spread pattern in two geographically distinct areas.

For the 2020 timeline, we saw an overall low number of confirmed and death cases in OD as compared to NY, where we identified a higher prevalence of infection and deaths. This is evident from the incidence rates calculated for both the states. We found high confirmed cases for the age group 15–60 years and higher deaths in the age group above 60 years for both states as well. Higher deaths in ≥60 years of age may be due to the predisposition of older cohorts to comorbidities [39]. Lifestyle choices and behavioral differences seem to be a leading factor when it comes to the higher case numbers among younger people [20]. However, the infection rate between males and females was particularly discriminatory in OD rather than NY. The higher death numbers in males correlate with previous studies conducted in China and Italy [40]. Factors such as the high prevalence of smoking rates in males [41] and stronger innate immune responses in females can be linked to less risk of infection in females than in males [41,42]. The natural vulnerability of men to COVID-19 can also be explained by the presence of a high concentration of ACE2 in the circulating plasma of males than in females [15,18,40,43,44,45,46,47,48,49,50,51]. Lifestyle and work-culture differences may also explain the reduced cases among females in OD.

Studies have highlighted the strong impact of meteorological factors on viral as well as host susceptibility to SARS-CoV-2 [52,53,54,55,56,57,58,59]. We recognized the temperature-dependence of SARS-CoV-2 transmission, where the state of Odisha experienced a delay in COVID-19 progression from March to May 2020, when average temperatures were high, ranging from 28.2 to 31.4 °C (Figure 2A). According to our combined yearlong epi-curve (Figure 3), it is clearly established that the first wave in OD started to rise from June onward, peaked in September, and declined by November 2020. OD had a lower average temperature range of 23.7–29.9 °C during the entirety of the first wave. An increasing number of studies have correlated the high transmissibility and persistence of SARS-CoV-2 with low temperature and humidity [16,19,20,22,60]. The temperature-sensitive nature of the virus was also noticeable in NY in 2020, where confirmed case numbers were very prominent during March and April with an average temperature range of 6–12.5 °C, declining by May 2020 as the average temperature started to increase (Figure 2B). This is also evident from the first COVID-19 wave in NY illustrated in Figure 3.

Surprisingly, the 2021 timeline saw a vast expansion in case numbers in OD as compared to NY (Table 2). The overall case numbers in OD for 2021 were roughly 197 times higher than in 2020 (Table 1) during the specified period for our study. This is also evident from a sharp rise in incidence rate (Results, Section 3) and coincides with the beginning of the second wave in OD (Figure 3). Nevertheless, we did not observe any temperature effect on SARS-CoV-2 transmission in OD since its rapid rise from April 2021 through May 2021, when average temperatures were between 30.4 and 31.4 °C (Figure 2A). This observation is rather contrasting with the 2020 timeline for OD. This can be possibly explained by the emergence of coronavirus variants in OD, especially following the onset of Delta variant cases in India from January 2021 [61,62]. The Delta Plus variant is a sublineage of the existing Delta variant of concern and had been first identified in India during April 2021 and had spread across 16 states in India and 9 other countries globally [63]. There was, however, a sheer dominance of Delta variant cases such that the proportion of cases with Delta rose from 21% in March 2021 to 98% in July 2021 in OD; currently spread across 30 districts within OD [64]. Hence, the second wave in OD can be attributed to the coronavirus Delta variants with increased adaptive advantages such as high pathogenicity, transmissibility, and enhanced immune escape properties compromising vaccine efficiency [65,66,67,68,69,70,71]. However, temperature sensitivity was observed for NY when the second wave started from November 2020, declining by April 2021, with a lower average temperature range of 0.5–12.5 °C (Figure 2B). From the yearlong epidemiological curve, there were three major observations: (1) We can visualize the precise onset and decline for the first and the second wave for COVID-19 in both the states. (2) For the first wave, OD had a delayed onset with fewer confirmed cases compared to the rapid progression in NY with higher confirmed case numbers. (3) For the second wave, OD had a very high number of confirmed cases, which was not only higher than that in NY but also higher than that in OD during the first wave. We could further sense a seasonal activity of the SARS-CoV-2 virus from the epi-curve. This is consistent with past findings, which determined that COVID-19 infection is seasonally linked due to the temperature-sensitive nature of the virus [22].

The duration of COVID-19 first wave (June–November 2020) and second wave (April 2021–ongoing) coincides with the peak activity of influenza virus that affects the Indian subcontinent during monsoon (June–October) [38]. Similarly, the first (March–May 2020) and second (November 2020–April 2021) wave in NY harmonized with the peak seasonal activity of influenza in the United States from December to February and could last as late as May [37].

Despite the inclusion of a large number of case data, which poses as a strength, our study had some limitations. First, we divided the timelines for 2020 and 2021 due to restricted access to public domain servers. Second, we could not gather age- and gender-specific datasets for the state of Odisha and New York in 2021, with 37.5% missing information in 2020. This highlights the urgency for the development of an efficient epidemiological database to track COVID-19 information in India on a daily basis.

Several other factors may contribute to our overall observation of COVID-19 incidences in OD and NY. It is clear from earlier research that, in addition to temperature, humidity plays an essential part in COVID-19 transmission. Past studies collectively agree that temperature and humidity inversely correlate with SARS-CoV-2 transmission in association with the persistence of airborne particles under low-temperature and low-humidity conditions [22]. Two particular studies highlight the correlation of absolute humidity to COVID-19 spread in Japan, China, England, and Germany [72,73]. Even though the studies found a correlation in the spread and decay of the COVID-19 pandemic with absolute humidity in four different countries, the authors additionally address that the contribution of the said factor could vary across different regions.

Population density also contributes to a higher risk of SARS-CoV-2 infection. Mounting evidence suggests a positive correlation of population density with increased COVID-19 spread and incidence within different geographical areas [22,73,74]. Population density may have contributed to the slow spread in OD during the first wave when social distancing and lockdown (24 March 2020–31 May 2020) protocols were heavily implemented by government officials [75]. Overall, the contribution of population density toward COVID-19 cases remains around 30–50% in countries such as Japan, China, England, and Germany [73].

Similarly, the low overall case fatality rate in OD for 2020 and 2021 could be explained by adherence to lockdown protocol, lifestyle choices, and, more importantly, vaccination status. OD has been in a privileged position owing to the free-of-cost access to indigenous vaccines manufactured in India [76], with efforts to finish the entire vaccination drive by the end of this year [77]. Although vaccine development started before the initiation of the second wave in OD, the new reports of emerging variants of concern (January 2021), especially the Delta variant may challenge the efficacy of vaccines. As discussed earlier, the Delta variant could be an important confounding factor in the unexpected rise of case numbers in the state of Odisha during 2021. The drastic reduction of case fatality in NY from 2020 to 2021 could also be explained by compliance with government-implemented containment strategies and effective vaccination policy, which can be correlated to factors such as temperature, humidity, population density, behavior, and emerging SARS-CoV-2 variants.

## 5. Conclusions

COVID-19 outbreak serves as a remarkable example of animal–human interface infection. The available epidemiological datasets and information during such an outbreak are equally important for evaluating the risks of viral transmission and start countermeasure protocols. Such information includes daily confirmed cases and deaths, case fatality, incidence rate, and also, how different regions have been affected, managing situations evenly with steady vaccination drives, and correlating information available from past outbreaks. Combining this information and a better understanding of the outcome is paramount to refine the risk assessment as the outbreak continues.

We report the transmission dynamics of COVID-19, encompassing the first and second waves in OD and NY, two geographically isolated places. Our data can disseminate valuable information to the scientific community, especially in the wake of a third wave. We observe an interesting age, gender, and temperature bias of the virus during the initial half of the pandemic in OD. As the pandemic progressed, new variants started to emerge. However, it is not necessary that the huge case number in OD is only due to SARS-CoV-2 variants, large population densities with behavioral factors such as travel, failure to use mask and social distancing, and more importantly, insufficient vaccination have become prominent factors during the second wave in OD. High confirmed case numbers were dominating the state of Odisha, India, even after May 2021.

As the outbreak of the novel SARS-CoV-2 and its emerging variants becomes rapidly dominant, we are on the brink of a much anticipated third wave. An intensified vaccination drive along with continuous local epidemiological monitoring and genomic surveillance is required to contain the threat posed by the COVID-19 pandemic. We ought to be vigilant, practice precautionary measures, and get vaccinated immediately to restrict the possible third wave in Odisha and the rest of the world.

## Figures and Tables

**Table 1:** Baseline characteristics for confirmed and death cases of COVID-19 as well as case fatality rates and crude death rates in the state of New York and Odisha through 31 May 2020. A total of 203,006 confirmed and 17,766 death cases were reported in NY, and 2223 confirmed and 7 death cases were reported in OD. The ‘Before March 2020’ row includes cumulative data of January and February 2020. Percentages are calculated in parenthesis under confirmed and death case columns. The ‘Missing’ row under the age group column indicates confirmed and death cases without the identification of the correct age group it falls under. The ‘Unknown’ row marked under the gender distribution column indicates confirmed case and death numbers lacking information of the gender category it falls under. Abbreviations: OD, State of Odisha, India. NY, State of New York, USA.

Baseline Characteristics	Confirmed Cases *n* (%)		Death Cases *n* (%)		Case Fatality Rates (%)		Crude Death Rates per 100,000 of the Population (%)	
	OD	NY	OD	NY	OD	NY	OD	NY
Overall/Total	2223	203,006	7	17,766	0.3	8.8	0.015	91.175
Age group, years 0–14 15–40 41–60 60+ Missing								
	57 (2.6) 1317 (59.2) 492 (22.1) 82 (3.7) 275 (12.4)	4955 (2.4) 69,610 (34.3) 69,402 (34.2) 46,021 (22.7) 13,018 (6.4)	0 (0) 2 (0.15) 0 (0) 5 (6.09) 0 (0)	10 (0.2) 625 (0.8) 3556 (5.1) 11,694 (25.4) 1881 (14.4)	0.0 0.2 0.0 6.1 0.0	0.2 0.9 5.1 25.4 14.4	0.0 0.004 0.0 0.011 0.0	0.051 3.208 18.249 60.014 9.653
Gender-wise Male Female Unknown								
	1702 (76.6) 246 (11.1) 275 (12.4)	98,038 (48.3) 92,228 (45.4) 12,740 (6.3)	7 (0.4) 0 (0) 0 (0)	9634 (9.8) 6223 (6.7) 1909 (14.9)	0.4 0.0 0.0	9.8 6.7 15	0.030 0.0 0.0	101.841 62.088 9.798
Period (date of onset) Before March 2020 1–31 March 2020 1–30 April 2020 1–31 May 2020								
	0 (0.0) 5 (0.2) 148 (6.7) 2070 (93.1)	1 (0.0) 65,203 (32.1) 109,342 (53.9) 28,460 (14)	0 (0.0) 0 (0.0) 1 (14.3) 6 (85.7)	0 (0.0) 2193 (12.3) 12,742 (71.7) 2831 (15.9)	- 0.0 0.7 0.3	0.0 3.4 11.7 9.9	0.0 0.0 0.002 0.013	0.0 11.255 65.392 14.529

**Table 2:** Baseline characteristics for confirmed and death cases of COVID-19 as well as case fatality rates and crude death rates in the state of New York and Odisha from 1 March 2021 to 31 May 2021. A total of 159,364 confirmed and 3570 death cases were reported in NY, and 436,455 confirmed and 875 death cases were reported in OD. The ‘Overall/Total’ row includes the cumulative data from 1 March 2021 to 31 May 2021. The ‘Before March 2021’ row includes cumulative data of January and February 2021. Abbreviations: OD, State of Odisha, India. NY, State of New York, USA.

Baseline Characteristics	Confirmed Cases n (%)		Death Cases n (%)		Case Fatality Rates (%)		Crude Death Rates per 100,000 of the Population (%)	
	OD	NY	OD	NY	OD	NY	OD	NY
Overall/Total (1 March–31 May 2021)	436,455	159,364	875	3570	0.2	2.2	1.84	17.50
Age group and gender distribution	*** Case data could not be retrieved ***							
Period (date of onset) Before March 2021 1–31 March 2021 1–30 April 2021 1–31 May 2021								
	7411 4034 113,296 319,125	223,871 91,966 54,294 13,104	40 5 133 737	4192 1782 1232 556	0.5 0.1 0.1 0.2	1.9 1.9 2.3 4.2	0.08 0.01 0.28 1.55	20.55 8.74 6.04 2.73

## Abbreviations

ACE2	Angiotensin-converting enzyme 2
CDC	Centers for Disease Control and Prevention
CDR	Crude death rate
CFR	Case fatality rate
COVID-19	Coronavirus disease 2019
MERS-CoV	Middle East respiratory syndrome coronavirus
NCIRD	National Center for Immunization and Respiratory Diseases
NY	State of New York
OD	State of Odisha
SARS-CoV	Severe acute respiratory syndrome coronavirus
SARS-CoV-2	Severe acute respiratory syndrome coronavirus 2
S-protein	Spike protein
USA	United States of America
VOC	Variants of concern
WHO	World Health Organization
